# Biochemical toxicity and DNA damage of imidazolium-based ionic liquid with different anions in soil on *Vicia faba* seedlings

**DOI:** 10.1038/srep18444

**Published:** 2015-12-17

**Authors:** Tong Liu, Lusheng Zhu, Jinhua Wang, Jun Wang, Jun Zhang, Xi Sun, Cheng Zhang

**Affiliations:** 1 College of Resources and Environment, Key Laboratory of Agricultural Environment in Universities of Shandong, Shandong Agricultural University, Taian, 271018, PR China

## Abstract

In the present study, the toxic effects of 1-octyl-3-methylimidazolium chloride ([Omim]Cl), 1-octyl-3-methylimidazolium bromide ([Omim]Br) and 1-octyl-3-methylimidazolium tetrafluoroborate ([Omim]BF_4_) in soil on *Vicia faba* (*V. faba*) seedlings at 0, 100, 200, 400, 600 and 800 mg kg^−1^ were assessed for the first time at the cellular and molecular level. Moreover, the toxicity of these three ionic liquids (ILs) was evaluated, and the influence of anions on the toxicity of the ILs was assessed. The results showed that even at 100 mg kg^−1^, the growth of *V. faba* seedlings was inhibited after exposure to the three ILs, and the inhibitory effect was enhanced with increasing concentrations of the three ILs. The level of reactive oxygen species (ROS) was increased after exposure to the three ILs, which resulted in lipid peroxidation, DNA damage and oxidative damage in the cells of the *V. faba* seedlings. In addition, the anion structure could influence the toxicity of ILs, and toxicity of the three tested ILs decreased in the following order: [Omim]BF_4_ > [Omim]Br > [Omim]Cl. Moreover, oxidative damage is the primary mechanism by which ILs exert toxic effects on crops, and ILs could reduce the agricultural productivity.

Ionic liquids (ILs) are a novel class of solvents that are entirely composed of ions at room temperature. ILs usually consist of large asymmetric organic cations, such as imidazolium, pyridinium, pyrrolidinium and quaternary ammonium, as well as small inorganic or organic anions, such as Cl^−^, Br^−^ and BF_4_^−1^. ILs can serve as new types of multi-functional green solvents because of their unique physicochemical properties, such as negligible vapor pressure, good solubility, thermostabilization and recyclability^1^,^2^. Therefore, ILs have unique properties that can be useful in different fields, such as organic synthesis, catalysis, electrochemical and separation processes^1^,^3^. Currently, the commercial availability of ILs is increasing.

The increased interest in the use of ILs in industry will inevitably result in environmental exposure through various channels, such as accidental spills, leaching of landfill sites and via effluents or irrigation^1^. Although the application of ILs as green solvents has increased, relatively little is known about their toxicity. Studies have shown that ILs exhibit broad toxicity, and the toxicity of ILs is even greater than that of traditional organic solvents^4^,^5^. Therefore, we must consider the potential risks of this new chemical to people and the environment.

In recent years, research assessing the toxicity of ILs has been a very rapidly growing discipline. A considerable amount of data has been published on the toxicity of ILs in aquatic and terrestrial environments^6^,^7^,^8^,^9^. The toxicity has been evaluated on the basis of test systems at different levels of biological complexity, from molecules and cells to organisms representing different trophic levels. However, it is worth noting that previous studies examining the toxic effects of ILs on plants were primarily performed in nutrient solutions. In the natural environment, plants are mainly grown in soil. Moreover, soil represents a more complicated environment in which to study the toxic effects of ILs on plants, especially the influence of ILs on crops. Although the toxic effects of ILs in soil on terrestrial plants have been previously studied, the only endpoint assessed was growth inhibition^10^,^11^,^12^,^13^,^14^,^15^. Therefore, an investigation of the toxic effects of ILs in soil on plants at the cellular and molecular levels is important to achieve a comprehensive understanding of the toxicity of ILs and their influence on the agricultural environment.

Imidazolium-based ILs are the most widely used ILs^16^,^17^. The imidazolium-based ILs 1-octyl-3- methylimidazolium chloride ([Omim]Cl), 1-octyl-3-methylimidazolium bromide ([Omim]Br) and 1-octyl-3-methylimidazolium tetrafluoroborate ([Omim]BF_4_) have been previously used to study the toxic effects of ILs on organisms^6^,^18^,^19^. Additionally, *V. faba* is one of the primary agricultural crops in China and around the world^20^,^21^. Moreover, *V. faba* is a good environmental indicator organism; thus, it is widely used to study the toxic effects of chemicals^22^,^23^. With these factors in mind, we chose to study the toxic effects of the three imidazolium-based ILs in soil on *V. faba* seedlings at the cellular and molecular levels for the first time. The purpose of the present study was to evaluate the toxicity of the three ILs and to ascertain the influence of anions on the toxicity of ILs. Moreover, the present results could provide a theoretical basis for evaluating the environmental safety of ILs in soil and the influence of ILs on agricultural production.

## Results

### Effects of [Omim]Cl, [Omim]Br and [Omim]BF_4_ on the growth of *V. faba* seedlings

**Figure 1:**
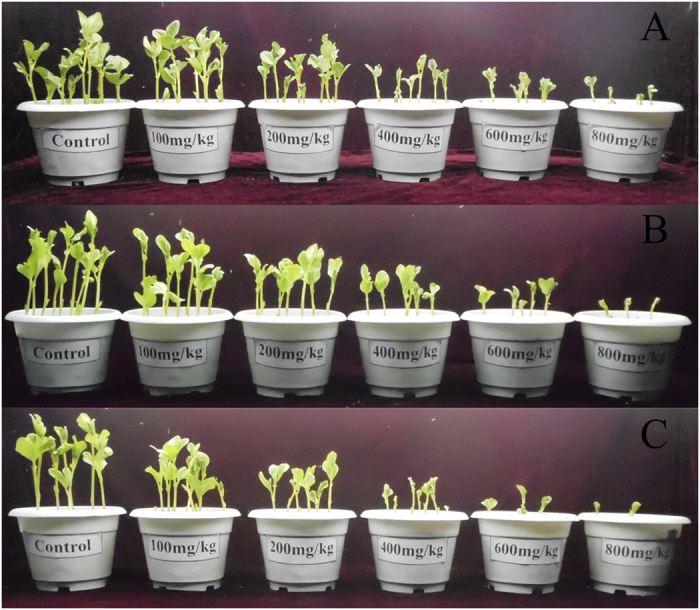
Influence of [Omim]Cl (**A**), [Omim]Br (**B**) and [Omim]BF_4_ (**C**) at concentrations of 0, 100, 200, 400, 600 and 800 mg kg^−1^ on the growth of *V. faba* seedlings. Images were obtained on the 10^th^ day of exposure.

As shown in Fig. 1, the growth of *V. faba* seedlings was significantly inhibited after exposure to the three ILs, and this growth showed similar trends during the exposure period. Moreover, the inhibitory effect was enhanced with increasing concentrations of the three ILs. At the highest concentration (800 mg kg^−1^), the *V. faba* seedlings treated with the three ILs nearly stopped growing.

As shown in Table 1, the shoot length, root length and dry weight significantly decreased with increasing concentrations of the three ILs. Significant differences in the shoot length, root length and dry weight were observed when the IL treatments were applied at 100 mg kg^−1^. The root length was inhibited more significantly than the shoot length and dry weight, and the inhibition induced by [Omim]BF_4_ was more obvious than that induced by [Omim]Cl and [Omim]Br. The roots of *V. faba* seedlings treated with [Omim]Cl and [Omim]Br stopped growing when these ILs were applied at 800 mg kg^−1^. Meanwhile, the roots of *V. faba* seedlings treated with [Omim]BF_4_ stopped growing when this IL was applied at 600 mg kg^−1^.

**Figure 2:**
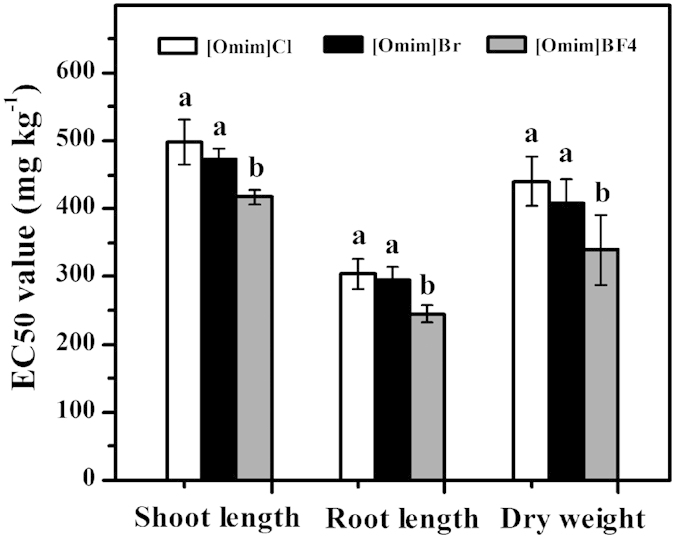
The EC_50_ values of [Omim]Cl, [Omim]Br and [Omim]BF_4_ with respect to their effects on shoot length, root length and dry weight of *V. faba* seedlings. The bars are the means ± standard error of three replicates. Different letters above the columns indicate significant differences (*p* < 0.05) between treatments as determined by the least significant difference (LSD) test.

The 50% effective concentration (EC_50_) values of the three ILs with respect to their effects on shoot length, root length and dry weight are listed in Fig. 2. The EC_50_ value for root length was significantly lower than that for shoot length and dry weight. For shoot length, root length and dry weight, the EC_50_ values of [Omim]BF_4_ were significantly lower than those of [Omim]Cl and [Omim]Br. For shoot length, root length and dry weight, the EC_50_ values of [Omim]Cl were 498 mg kg^−1^, 304 mg kg^−1^ and 440 mg kg^−1^, respectively; the EC_50_ values of [Omim]Br were 473 mg kg^−1^, 295 mg kg^−1^ and 408 mg kg^−1^, respectively; and the EC_50_ values of [Omim]BF_4_ were 417 mg kg^−1^, 245 mg kg^−1^ and 339 mg kg^−1^, respectively.

### Effect of [Omim]Cl, [Omim]Br and [Omim]BF_4_ on the pigment content

**Figure 3:**
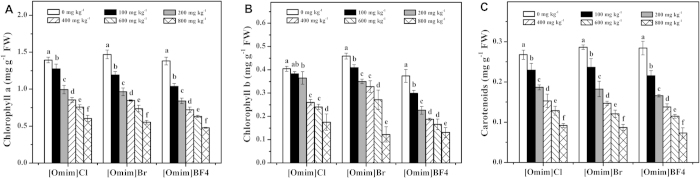
Effects of [Omim]Cl, [Omim]Br and [Omim]BF_4_ on the contents of chlorophyll a (**A**), chlorophyll b (**B**) and carotenoids (**C**) in *V. faba* seedling leaves. The bars are the means ± standard error of three replicates. Different letters above the columns indicate significant differences (*p* < 0.05) between treatments as determined by the least significant difference (LSD) test.

As shown in Fig. 3, the chlorophyll a, chlorophyll b and carotenoid contents showed similar trends during the exposure period and were significantly reduced after exposure to the three ILs. The changes in chlorophyll a and carotenoids were more significant than the changes in chlorophyll b. In general, significant differences in chlorophyll a, chlorophyll b and carotenoids were observed when the ILs were applied at 100 mg kg^−1^.

### Effect of [Omim]Cl, [Omim]Br and [Omim]BF_4_ on the generation rate of superoxide free radical (O_2_^·−^) and the content of hydrogen peroxide (H_2_O_2_)

**Figure 4:**
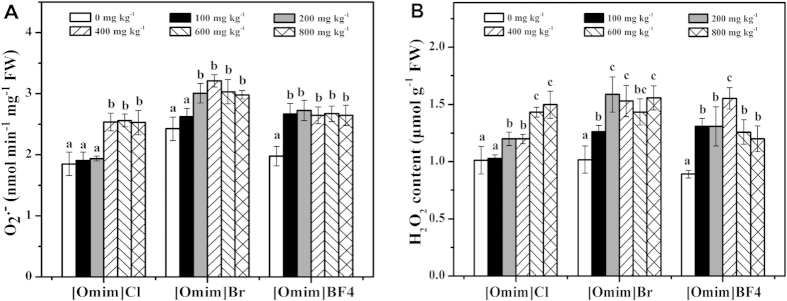
Effects of [Omim]Cl, [Omim]Br and [Omim]BF_4_ on the generation rate of O_2_^−^ (**A**) and the content of H_2_O_2_ (**B**) in *V. faba* seedling leaves. The bars are the means ± standard error of three replicates. Different letters above the columns indicate significant differences (*p* < 0.05) between treatments as determined by the least significant difference (LSD) test.

As shown in Fig. 4A, the O_2_^·−^ generation rate was significantly increased after exposure to the three ILs. The significant differences induced by [Omim]Cl, [Omim]Br and [Omim]BF_4_ were first observed at 400 mg kg^−1^, 200 mg kg^−1^ and 100 mg kg^−1^, respectively.

As shown in Fig. 4B, the content of H_2_O_2_ followed a trend similar to that of the O_2_^·−^ generation rate, as both H_2_O_2_ content and O_2_^·−^ generation rate increased with increasing concentrations of the three ILs. The significant differences induced by [Omim]Cl, [Omim]Br and [Omim]BF_4_ were first observed at 200 mg kg^−1^, 100 mg kg^−1^ and 100 mg kg^−1^, respectively.

### Effect of [Omim]Cl, [Omim]Br and [Omim]BF_4_ on the level of lipid peroxidation and the degree of DNA damage

**Figure 5:**
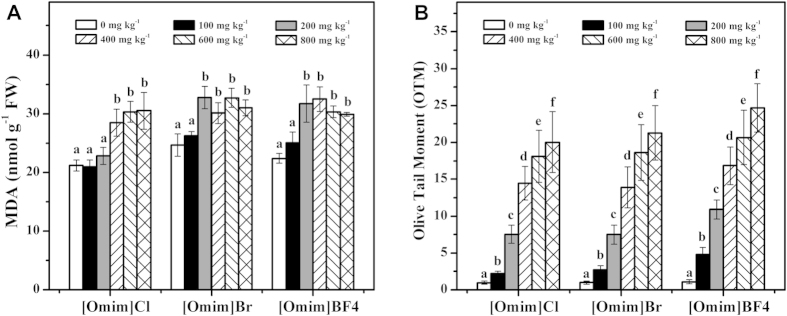
Effects of [Omim]Cl, [Omim]Br and [Omim]BF_4_ on the level of lipid peroxidation (**A**) and the degree of DNA damage (**B**) in *V. faba* seedling leaves. The bars are the means ± standard error of three replicates. Different letters above the columns indicate significant differences (*p* < 0.05) between treatments as determined by the least significant difference (LSD) test.

The malondialdehyde (MDA) contents of the *V. faba* seedlings exposed to [Omim]Cl, [Omim]Br and [Omim]BF_4_ are listed in Fig. 5A. At 100 mg kg^−1^, the three ILs had little impact on the MDA content. The toxic effects of the three ILs were significantly enhanced at 400 mg kg^−1^, 200 mg kg^−1^ and 200 mg kg^−1^, respectively. Although the MDA content of *V. faba* seedlings exposed to 600 mg kg^−1^ and 800 mg kg^−1^ [Omim]BF_4_ treatments showed a decreasing trend during the exposure period, the MDA contents remained significantly higher than those in the control.

As shown in Fig. 5B, the olive tail moment (OTM, the distance between the center of the head and the center of the tail and the percentage of total DNA in the tail) values were significantly enhanced after exposure to the three ILs, and significant differences were observed at 100 mg kg^−1^. At 100, 200, 400, 600 and 800 mg kg^−1^, the OTM values induced by [Omim]Cl were 2, 8, 15, 19 and 21 times higher, respectively, than the OTM value in the control; the OTM values induced by [Omim]Br were 2, 8, 14, 19 and 22 times higher, respectively, than the OTM value in the control; and the OTM values induced by [Omim]BF_4_ were 4, 10, 15, 19 and 22 times higher, respectively, than the OTM value in the control.

In addition, we studied the percentage of cells (n = 100) in different DNA damage classes. The present results showed that the cells in the control group were distributed in level I. When exposed to the three ILs at 100, 200 and 400 mg kg^−1^, the cells were primarily distributed in levels II, III and IV. When exposed to 600 and 800 mg kg^−1^ ILs, the cells were primarily distributed in level V.

### Effect of [Omim]Cl, [Omim]Br and [Omim]BF_4_ on superoxide dismutase (SOD) and catalase (CAT) activities

**Figure 6:**
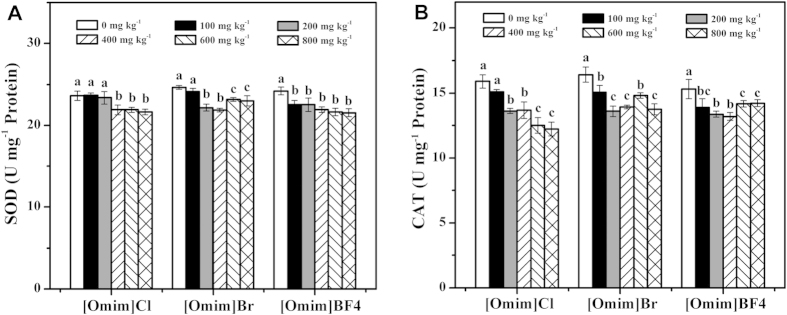
Effects of [Omim]Cl, [Omim]Br and [Omim]BF_4_ on the activities of SOD (**A**) and CAT (**B**) in *V. faba* seedling leaves. The bars are the means ± standard error of three replicates. Different letters above the columns indicate significant differences (*p* < 0.05) between treatments as determined by the least significant difference (LSD) test.

As shown in Fig. 6A, SOD activities were inhibited after exposure to the three ILs. In general, SOD activities decreased with increasing concentrations of the three ILs; however, this decrease was not observed for [Omim]Br at 600 and 800 mg kg^−1^. For [Omim]Cl, [Omim]Br and [Omim]BF_4_, significant differences were first observed at 400 mg kg^−1^, 200 mg kg^−1^ and 100 mg kg^−1^, respectively.

As shown in Fig. 6B, CAT activity showed a trend similar to that of SOD activity, as both CAT and SOD activities decreased after exposure to the three ILs. CAT activity decreased with increasing the concentrations of [Omim]Cl. Although the CAT activity showed an upward trend after exposure to [Omim]Br at 600 mg kg^−1^, it was still lower than that in the control group. CAT activities decreased after exposure to [Omim]BF_4_ at 100, 200 and 400 mg kg^−1^; however, at 600 and 800 mg kg^−1^, CAT activities increased.

### Effect of [Omim]Cl, [Omim]Br and [Omim]BF_4_ on ascorbic acid (ASA) and reduced glutathione (GSH) contents

**Figure 7:**
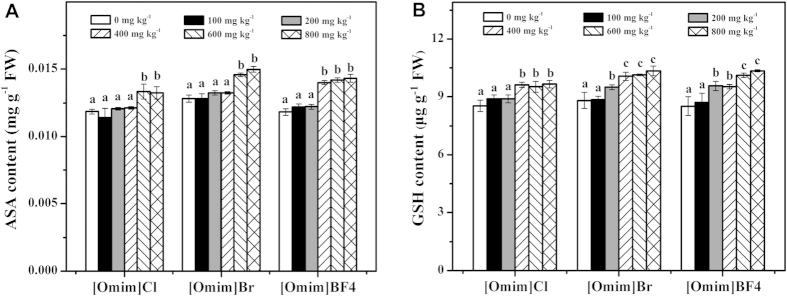
Effects of [Omim]Cl, [Omim]Br and [Omim]BF_4_ on the contents of ASA (**A**) and GSH (**B**) in *V. faba* seedling leaves. The bars are the means ± standard error of three replicates. Different letters above the columns indicate significant differences (*p* < 0.05) between treatments as determined by the least significant difference (LSD) test.

As shown in Fig. 7A, ASA contents were enhanced after exposure to the three ILs. However, the three ILs had little influence on the ASA contents when applied at 100 and 200 mg kg^−1^. When [Omim]Cl and [Omim]Br were applied at 600 and 800 mg kg^−1^, the ASA contents were significantly enhanced. For [Omim]BF_4_, significant differences were first observed at 400 mg kg^−1^.

As shown in Fig. 7B, the GSH content showed a trend similar to that of the ASA content, as both GSH and ASA contents were enhanced after exposure to the three ILs. For [Omim]Cl, the GSH contents remained stable at 400, 600 and 800 mg kg^−1^. For [Omim]Br and [Omim]BF_4_, the GSH contents increased as the concentrations increased. For [Omim]Cl, [Omim]Br and [Omim]BF_4_, significant differences were first observed at 400, 200 and 200 mg kg^−1^, respectively.

## Discussion

When plants are exposed to stress conditions, the most obvious phenomenon is the inhibition of growth^24^. In the present study, the growth of *V. faba* seedlings was significantly inhibited after exposure to [Omim]Cl, [Omim]Br and [Omim]BF_4_. Although the inhibitory effect was not obvious at low concentrations, it was significantly enhanced with increasing concentrations of the three ILs. The behavior of ILs is similar to that of plant hormones or salts at low concentrations, as ILs in small amounts have a minimal influence on the growth of plants^18^,^25^. However, at high concentrations, ILs could have a more significant impact on the membrane system by increasing lipophilicity and uptake, resulting in higher internal concentrations^11^. Thus, ILs in excess inevitably affect the growth of plants. In addition, the root of *V. faba* seedlings were the most significantly affected organ in the present study. We believe that this result is caused by long-term direct contact with ILs, which results in the excessive accumulation of ILs in roots. Previous reports also showed that the growth of plants is inhibited after exposure to ILs, regardless of whether exposure occurs in soil or nutrient solution^9^,^10^,^11^,^12^,^13^,^14^,^15^,^18^,^25^,^26^. Therefore, we believe that the mechanism by which ILs exert toxicity in different media is consistent.

In the present study, there were no differences between the EC_50_ values of [Omim]Cl and [Omim]Br for shoot length, root length and dry weight. However, the EC_50_ value of [Omim]BF_4_ differed from that of [Omim]Cl and [Omim]Br for shoot length, root length and dry weight. A similar result was also reported by Bubalo *et al.*^27^, who studied the toxic effects of [C_4_mim][CH_3_CO_2_], [C_4_mim]BF_4_ and [C_4_mim]Br in solution on barley seedlings. The EC_50_ values of the three ILs ranged from 300 mg L^−1^ to 600 mg L^−1^ for shoot length and root length, and there were significant differences among the EC_50_ values of [C_4_mim][CH_3_CO_2_], [C_4_mim]BF_4_ and [C_4_mim]Br for shoot length, root length and dry weight. A similar result was also reported by Matzke *et al.*^11^, who studied the role of anion species in the toxicity of 1-alkyl-3-methylimidazolium ILs. Therefore, we believe that the structures of the anions could influence the toxicity of the ILs. In addition, based on the EC_50_ values of the three ILs in the present study, we believe that these ILs can be ordered based on their toxicity as follows: [Omim]BF_4_ > [Omim]Br > [Omim]Cl.

Photosynthesis is the basis of plant growth and development, and chlorophyll is an important pigment that captures light energy from the sun to produce glucose via photosynthesis^28^. Carotenoids are other important pigments that can transfer energy to chlorophyll^29^. In the present study, the contents of chlorophyll a, chlorophyll b and carotenoids were significantly decreased after exposure to the three ILs. Therefore, we believe that the three ILs affected the photosynthesis of *V. faba* seedlings, resulting in an inhibitory effect on growth. Previous studies also reported that ILs could inhibit photosynthesis by inhibiting the activity of the Hill reaction, which is the first step of photosynthesis^18^. In addition, studies have shown that carotenoids belong to a group of lipophilic antioxidants and can prevent the formation of reactive oxygen species (ROS) to protect the photosynthetic apparatus^29^,^30^. Therefore, we believe that the decrease in carotenoid content is associated with the increased ROS level. Similar results were also reported by Liu *et al.*^9^, Liu *et al.*^26^ and Bubalo *et al.*^27^ who studied the toxic effects of ILs on plants.

Various environmental stresses can disrupt the balance of cellular homeostasis in plants, resulting in increased accumulation of ROS, including O_2_^·−^ and H_2_O_2_^28^,^29^,^31^. In the present study, the O_2_^·−^ generation rate and the H_2_O_2_ content were enhanced after exposure to the three ILs, indicating that the balance of generation and scavenging of ROS in *V. faba* seedlings was disrupted by the three ILs. In addition, the ROS level in [Omim]Br and [Omim]BF_4_ treatments stopped increasing at high concentrations, which may be caused by the defense of antioxidant system. Kumar *et al.*^32^,^33^ studied the toxic effects of ILs on green seaweed and found that the O_2_^·−^ generation rate and the H_2_O_2_ content were increased after exposure to ILs. Similar results were also reported by Liu *et al.*^9^, Bubalo *et al.*^27^ and Zhang *et al.*^34^ when they studied the toxic effects of ILs on plants.

Excess ROS are highly reactive and toxic, which can cause damage to proteins, lipids and DNA^29^,^35^. MDA is the byproduct of lipid peroxidation and reflects the degree of plant sensitivity to ROS-induced oxidation^36^. In the present study, the MDA content was also increased after exposure to the three ILs, which indicated that the three ILs caused lipid peroxidation in the cells of *V. faba* seedlings. Similar results were also obtained by Liu *et al.*^9^, Liu *et al.*^26^, Bubalo *et al.*^27^ and Zhang *et al.*^34^ in their studies of the toxic effects of ILs on plants.

DNA is a nucleic acid that carries cellular genetic information. Comet assay was used to detect the degree of DNA damage, which indicates whether organisms have suffered genotoxic effects due to environmental stress^37^. Studies have shown that cellular DNA is damaged by excess ROS under various environmental stresses, such as high salt, ultraviolet radiation or exposure to toxic contaminants^23^,^38^,^39^. In the present study, the OTM values increased significantly after the *V. faba* seedlings were exposed to the three ILs. Moreover, the degree of DNA damage also increased with increasing concentrations of the three ILs. Therefore, we believe that the three ILs were genotoxic to the cells of *V. faba* seedlings. In addition, although the ROS level stopped increasing at high concentrations, the OTM values still increased. The reason is due to DNA repair occurs more slowly than DNA damage. The reduced ROS level resulted in the increase rate of OTM observed at concentrations ranging from 400 to 800 mg kg^−1^ was lower than that observed at concentrations ranging from 100 to 400 mg kg^−1^. The DNA of plants was seriously damaged by ILs also reported by Kumar *et al.*^32^,^33^, who studied the toxic effects of ILs on green seaweed.

Antioxidant enzymes such as SOD and CAT play an important role in scavenging the excess ROS in plant cells. SOD is considered to be the first line of defense against ROS, as it can catalyze the conversion of O_2_^·−^ to O_2_ and H_2_O_2_^40^. Nonetheless, H_2_O_2_ is still harmful to cells, and it must be scavenged by other antioxidant enzymes, such as CAT. In the present study, SOD and CAT activities were inhibited after exposure to the three ILs, resulting in increases in the O_2_^·−^ generation rate and the H_2_O_2_ content. The SOD and CAT activities decreased after exposure to ILs was also reported by Liu *et al.*^18^, who studied the toxic effects of [Omim]Cl on rice seedlings.

Under environmental stress, the content of antioxidant compounds, such as ASA and GSH, also changed in response to oxidative stress. ASA is considered to be a powerful antioxidant compound due to its ability to donate electrons in a number of enzymatic and nonenzymatic reactions^41^. GSH can detoxify xenobiotic and prevent oxidative damage from ROS^42^. Moreover, ASA and GSH have a synergistic effect in the elimination of O_2·_^−^ and H_2_O_2_ via the ASA-GSH cycle. In the present study, although the activities of antioxidant enzymes were inhibited, it is likely that antioxidant compounds played an important role in removing excess ROS. Therefore, the contents of ASA and GSH in *V. faba* seedling leaves were increased after exposure to the three ILs, especially at 600 and 800 mg kg^−1^. Accordingly, the ROS level stopped increasing at high concentration treatments. Similar changes in the contents of antioxidant compounds after exposure to ILs were also reported by Ma *et al.*^19^ and Yu *et al.*^43^ in their studies of the toxic effects of [Omim]Br on *Daphnia magna* and snails.

**Figure 8:**
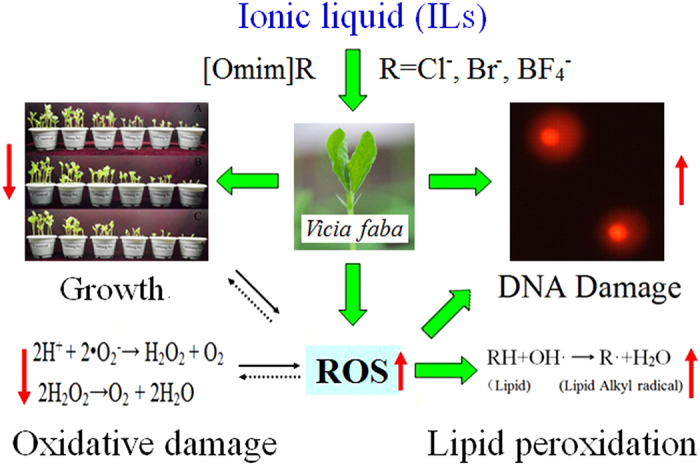
The relationship between these biomarkers used in the present study. The letter R represents the anions Cl^−^, Br^−^ and BF_4_^−^.

The relationship between these biomarkers was listed in Fig. 8. In the present study, the ROS level in *V. faba* seedlings was significantly increased after exposure to [Omim]Cl, [Omim]Br and [Omim]BF_4_. Excess ROS caused damage to the cells of *V. faba* seedlings, which resulted in lipid peroxidation, DNA damage and oxidative damage. Additionally, the excess ROS caused a decrease in pigment contents, which influenced photosynthesis and inhibited the growth of *V. faba* seedlings. On the other hand, the excess ROS was scavenged by antioxidant enzymes and antioxidant compounds, resulting in the ROS level return to normal. However, if the ROS level reaches a death threshold after exposure to high concentrations of ILs, it can not only result in damage to cells but also trigger death signaling^44^. Therefore, we believe that oxidative damage is the main mechanism by which ILs exert toxicity and that ILs can reduce agricultural productivity. Moreover, previous studies examining the toxic effects of ILs on plants also reported that ILs could inhibit plant growth and cause oxidative stress in plant cells^9^,^25^,^26^,^27^,^32^,^33^,^34^.

## Materials and Methods

### Materials

**Figure 9:**
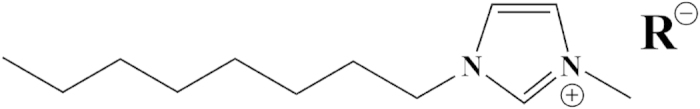
The structure of the ILs used in the present study. The letter R represents the anion (i.e., Cl, Br or BF_4_).

The ILs 1-octyl-3-methylimidazolium chloride, 1-octyl-3-methylimidazolium bromide and 1-octyl-3-methylimidazolium tetrafluoroborate ([Omim]Cl, [Omim]Br and [Omim]BF_4_; CAS NO. 64697-40-1, 61545-99-1 and 244193-52-0, respectively, all 99% purity) were obtained from Chengjie Chemical Co. Ltd. (Shanghai, China). The structure of the tested ILs is listed in Fig. 9.

The *V. faba* seeds were purchased from the College of Life Science, Shandong Agricultural University (Taian, China).

The tested soil was taken from the test field of Shandong Agricultural University (Taian, China) and sampled from a depth of 2-20 cm. The soil was filtered through a 2-mm sieve and then naturally air-dried in the laboratory. The physical and chemical characteristics of the tested soil were measured according to the method described by Wang *et al.*^45^. The organic matter content, available potassium content, organic nitrogen content, available phosphorus content, maximum field capacity and pH of the soil were 17.6 ± 1.1 g kg^−1^, 125.7 ± 7.4 mg kg^−1^, 132.3 ± 9.6 mg kg^−1^, 18.4 ± 1.6 mg kg^−1^, 18.5 ± 1.4% and 7.6 ± 0.4, respectively.

### Experimental design

Until now, no reports have focused on the toxic effects of [Omim]Cl, [Omim]Br and [Omim]BF_4_ in soil on plants. Therefore, the following concentrations were established to study the effects of these three ILs on the growth of *V. faba* seedlings: 0, 0.1, 1, 5, 10, 50, 100, 200, 400, 600, 800 and 1000 mg kg^−1^. The results of preliminary experiments showed that the growth conditions of *V. faba* seedlings at 0, 0.1, 1, 5, 10, 50 and 100 mg kg^−1^ were consistent. However, the *V. faba* seedlings stopped growing at 1000 mg kg^−1^. Therefore, based on these preliminary experiments, the concentrations used for testing were 0, 100, 200, 400, 600 and 800 mg kg^−1^.

A total of 8 g of each IL was dissolved in 500 mL of deionized water. Subsequently, the appropriate amounts of [Omim]Cl, [Omim]Br and [Omim]BF_4_ solutions and deionized water were added to 1000 g of soil such that the moisture of the soil was controlled at 60%. For each IL, eighteen pots were prepared and divided into six groups according to the IL concentration. Next, the soil was mixed to sufficiently homogenize the [Omim]Cl, [Omim]Br and [Omim]BF_4_ and was transferred into pots.

*V. faba* seeds were selected and surface sterilized in 30% sodium hypochlorite solution for 10 min, they were then thoroughly washed with distilled water. The sterilized seeds were soaked in distilled water for 24 h, and the water was replaced every 8 h. Then, ten seeds that were approximately the same size were selected and sown at a maximum depth of 1 cm from the surface. The seeds were vertically inverted in the pots, with the endosperm of the seeds facing downward. The *V. faba* seeds were then cultivated in a temperature-controlled greenhouse at 22°C for 14 h/day and 18°C for 10 h/night. The light intensity in the greenhouse was 200 μmol m^−2^ s^−1^, and the relative humidity of the soil was monitored by weighing every second day and controlled at 50–60%. On the 10^th^ day of cultivation, the *V. faba* seedlings were randomly sampled for the various analyses.

### Determination of shoot length, root length and dry weight

On the 10^th^ day of cultivation, five *V. faba* seedlings from each replicate were randomly selected to measure the shoot length and root length according to the method of Wang *et al.*^25^. Subsequently, these *V. faba* seedlings were oven-dried at 105°C for 15 min and then at 65°C for 48 h to measure the dry weight using a 1/10,000 analytical balance^9^.

### Determination of pigment content

The chlorophyll a, chlorophyll b and carotenoid contents were measured according to the method described by Meloni *et al.*^46^. Fresh leaves (0.1 g) were randomly collected from a *V. faba* seedling in each replicate, and then the leaves were placed in a 15-mL centrifuge tube. Then, 10 mL of 80% acetone was added, and the tube was placed in the dark for 40 h. The pigment was extracted into the acetone, and the absorbance values of the resulting extracts were measured at 470 nm, 646 nm and 663 nm using an ultraviolet/visible spectrophotometer (Shimadzu, UV-2550). The results are expressed as mg g^−1^ fresh weight (FW) of seedlings.

### The generation rate of O_2_^·−^

The O_2_^·−^ generation rate was measured based on the method of Qiu *et al.*^47^ by monitoring nitrite formation. Fresh leaves (0.25 g) of *V. faba* seedlings from each replicate were ground with 2.5 mL of 50 mM phosphate buffer (pH 7.8) containing 1 mM ethylenediamine tetraacetic acid (EDTA) and 1% (w/v) polyvinylpyrrolidone (PVP). The extract was centrifuged at 10,800 rpm for 10 min, and subsequently, 50 mM phosphate buffer (0.5 mL, pH 7.8) and 1 mM hydroxylamine hydrochloride (1 mL) were added to the supernatant (0.5 mL). The mixture was incubated in a water bath at 25°C. Approximately 1 h later, 7 mM α-naphthylamine (1 mL) and 17 mM sulfanilamide (1 mL) were added to the sample, and the sample was incubated at 25°C for 20 min. The absorbance was then measured at 530 nm using an ultraviolet/visible spectrophotometer (Shimadzu, UV-2550). An NO_2_^−^ standard curve was used to calculate the generation rate of O_2_^·−^.

### Determination of the H_2_O_2_ content

The H_2_O_2_ content was determined according to the method of Qiu *et al.*^47^. For each replicate, 0.25 g of fresh leaves were homogenized in 2.5 mL of 0.1% (w/v) trichloroacetic acid (TCA) and centrifuged at 13,200 rpm for 15 min at 4°C. Then, 1 mL of the supernatant was added to 1 mL of 100 mM phosphate buffer (pH 7.0) and 2 mL of 1 M potassium iodide (KI). The absorbance of the mixture was measured at 390 nm. The H_2_O_2_ content was calculated based on a standard curve and is expressed as μmol g^−1^ FW.

### Measurement of lipid peroxidation

The lipid peroxidation level was determined by measuring the amount of MDA according to the method of Song *et al.*^48^. MDA was extracted from *V. faba* seedling leaves with 0.1% TCA solution, and then the sample was centrifuged at 10,800 rpm for 20 min. Next, an equal volume of 0.5% (w/v) thiobarbituric acid (TBA) in 20% TCA was added to the supernatant. The sample was incubated in a water bath (95°C) for 30 min, and then the samples were placed in an ice bath to stop the reaction. Approximately 10 min later, the mixture was centrifuged at 10,800 rpm for 15 min, and the absorbance of the mixture was measured at 532 nm and 600 nm. The concentration of MDA is expressed as nmol g^−1^ FW.

### Comet assay

A comet assay was performed according to the method of Song *et al.*^23^. The extraction buffer (pH 7.5) containing 10 mM MgSO_4_, 50 mM KCl, 5 mM 4-hydroxyethyl piperazine ethyl sulfonic acid (HEPES), 0.3 mM dithiothreitol (DTT) and 0.25% (v/v) Triton X-100 was prepared according to the method of Lee and Lin^49^. Nuclei from *V. faba* seedling leaf cells were collected in 250 μL of cold extraction buffer using a new razor blade. Then, 100 μL of 0.8% normal melting agarose was pipetted onto microscope slides, one side of which was fully frosted. After solidification on ice, the second layer (40 μL of nuclear suspension solution mixed with 40 μL of 1% low-melting-point agarose) and third layer (50 μL of 0.5% low-melting-point agarose) were successively pipetted onto the microscope slides. The microscope slides were incubated in electrophoresis buffer (pH > 13), which contained 300 mM NaOH and 1 mM ethylenediamine tetraacetic acid disodium salt (Na_2_-EDTA), for 20 min. Subsequently, the slides were electrophoresed at 25 V (300 mA) for 15 min. Following electrophoresis, the slides were neutralized in 0.4 M Tris buffer (pH 7.5) for 15 min, and then stained with 30 μL of ethidium bromide (EB; 13 μg mL^−1^) for 15 min. Finally, the microscope slides were analyzed using a computerized image analysis system attached to a fluorescence microscope (Olympus, BX71). The OTM was used to quantify the extent of DNA damage.

### Determination of antioxidant enzyme activities

Fresh *V. faba* seedling leaves (0.5 g) were homogenized in 5 mL of extraction solution containing 50 mM ice-cold phosphate buffer (pH 7.8), 1 mM EDTA and 1% PVP. The sample was centrifuged at 13,200 rpm for 20 min at 4°C, and subsequently, the supernatant was used to assay protein content as well as SOD and CAT activities.

Protein content was measured according to the method of Bradford^50^ and standardized to bovine serum albumin. The absorbance was measured at 595 nm.

SOD activity was determined according to the method of Song *et al.*^23^. The reaction system (3 mL) contained 50 mM phosphate buffer (pH 7.8), 750 μM NBT, 130 mM methionine, 20 μM riboflavin, 0.1 mM EDTA, and 50 μL of the enzyme extract. After illumination at 5000 lx for 15 min, the absorbance of the mixture was measured at 560 nm using an ultraviolet/visible spectrophotometer (Shimadzu, UV-2550).

CAT activity was measured according to the method of Song *et al.*^48^. The reaction system (3 mL) contained 100 mM potassium phosphate buffer (pH 7.0), 20 mM H_2_O_2_ and 0.1 mL of the enzyme extract. The decrease in absorbance at 240 nm was recorded for 1 min using an ultraviolet/visible spectrophotometer (Shimadzu, UV-2550).

### Determination of antioxidant compound contents

Fresh *V. faba* seedling leaves (0.5 g) were homogenized in 5 mL of 5% TCA and centrifuged at 13,200 rpm for 10 min at 4°C. The supernatant was used to assay ASA and GSH contents.

The ASA content was measured according to the method of Shen *et al.*^51^. The reaction system (3 mL) contained 200 μL of the supernatant, 100 mM potassium phosphate buffer (pH 7.4), 44% H_3_PO_4_, 10% TCA (w/v), 4% 2,2-dipyridyl (w/v), 3% FeCl_3_ (w/v) and deionized water. The absorbance of the mixture was measured at 525 nm after a 1-h incubation at 37°C.

The GSH content was measured according to the method of Qiu *et al.*^47^. The reaction system (3 mL) contained 200 μL of the supernatant, 100 mM potassium phosphate buffer (pH 7.7) and 2.51 mg mL^−1^ 5,5′-dithiobis (2-nitrobenzoicacid) (DTNB). The absorbance of the mixture was measured at 412 nm after a 5-min incubation at 30°C.

### Statistical analysis

All analyses were performed in triplicate, and the results are presented as the mean ± SD. The data were analyzed using SPSS software (version 17.0, SPSS Inc.). One-way analysis of variance (ANOVA) was performed on all data and probit analysis was used to calculate the EC_50_ values. Differences among the treatments were assessed using the least significant difference (LSD) test at the *p* < 0.05 level. The Comet Assay Software Project (CASP) was used to analyze the comet images. The cells (n = 100) were categorized into grades of damage based on the percentage of tail DNA according to the method of Anderson *et al.*^52^ as follows: I, zero or minimal (<10%) tail DNA; II, low level of damage (10–25%); III, intermediate level of damage (25–50%); IV, high level of damage (50–75%); and V, extreme damage (>75%).

## Additional Information

**How to cite this article**: Liu, T. *et al.* Biochemical toxicity and DNA damage of imidazolium-based ionic liquid with different anions in soil on *Vicia faba* seedlings. *Sci. Rep.* **5**, 18444; doi: 10.1038/srep18444 (2015).

## Figures and Tables

**Table 1:** Effects of [Omim]Cl, [Omim]Br and [Omim]BF_4_ on the shoot length, root length and dry weight of *V. faba* seedlings.

IL	Concentration (mg kg^−1^)	Biomarkers		
		Shoot length (cm)	Root length (cm)	Dry weight (mg)
[Omim]Cl	0	18.93 ± 0.39^a^	9.67 ± 0.18^a^	254.96 ± 13.50^a^
	100	17.94 ± 0.33^b^	7.61 ± 0.28^b^	232.03 ± 10.22^b^
	200	15.45 ± 0.52^c^	5.35 ± 0.43^c^	194.57 ± 5.65^c^
	400	11.27 ± 0.06^d^	2.90 ± 0.23^d^	131.28 ± 7.75^d^
	600	8.12 ± 0.13^e^	0.67 ± 0.12^e^	94.67 ± 4.82^e^
	800	5.77 ± 0.12^f^	0^f^	73.23 ± 4.26^f^
[Omim]Br	0	20.60 ± 0.45^a^	10.62 ± 0.37^a^	281.59 ± 17.87^a^
	100	18.79 ± 0.37^b^	8.75 ± 0.47^b^	247.49 ± 21.96^b^
	200	15.89 ± 0.20^c^	6.09 ± 0.19^c^	205.40 ± 5.77^c^
	400	11.79 ± 0.09^d^	3.23 ± 0.29^d^	139.05 ± 4.08^d^
	600	7.63 ± 0.12^e^	0.94 ± 0.18^e^	97.67 ± 0.80^e^
	800	5.28 ± 0.12^f^	0^f^	75.66 ± 2.36^f^
[Omim]BF_4_	0	19.78 ± 0.27^a^	10.47 ± 0.24^a^	260.89 ± 9.16^a^
	100	16.32 ± 0.13^b^	7.71 ± 0.23^b^	209.83 ± 8.90^b^
	200	13.53 ± 0.22^c^	4.99 ± 0.15^c^	165.87 ± 1.10^c^
	400	9.32 ± 0.19^d^	1.39 ± 0.15^d^	113.59 ± 3.97^d^
	600	6.47 ± 0.15^e^	0^e^	85.03 ± 2.10^e^
	800	4.91 ± 0.05^f^	0^e^	62.07 ± 0.90^f^

Note: Different letters indicate significant differences between treatments at the *p* < 0.05 level.
